# Hepatoprotective effect of *Holothuria leucospilota* methanolic extract on dimethyl nitrosamine–induced hepatotoxicity in rats

**DOI:** 10.1002/ame2.12451

**Published:** 2024-07-01

**Authors:** Fatemeh Dejan, Amineh Daneshi, Javad Rajabi Aslani, Nasrollah Ahmadi, Narges Eskandari Roozbahani, Elaham Rahmanian, Reza Behmanesh, Hamid Reza Gheisari

**Affiliations:** ^1^ Department of Basic Sciences, School of Veterinary Medicine Shiraz University Shiraz Iran; ^2^ Department of Education Farzanegan High School Tehran Kohgiluyeh and Boyer Ahmad Iran; ^3^ Food Hygiene Department, School of Veterinary Medicine Shiraz University Shiraz Iran; ^4^ Department of Pathology, School of Veterinary Medicine Shiraz University Shiraz Iran; ^5^ Clinical Research Development Center, Imam Reza Hospital Kermanshah University of Medical Sciences Kermanshah Iran; ^6^ Molecular Dermatology Research Center Shiraz University of Medical Sciences Shiraz Iran; ^7^ Department of Anatomy, School of Medicine Shiraz University of Medical Sciences Shiraz Iran; ^8^ Faculty of Veterinary Medicine, Garmsar Branch, Department of Radiology Islamic Azad University Garmsar Iran

**Keywords:** dimethyl nitrosamine, hepatotoxicity, *Holothuria leucospilota*, sea cucumber

## Abstract

**Background:**

Complementary medicine is an interesting field for extracting bioactive compounds from various plant and animal sources. The hepatoprotective effect of the methanolic extract of a species of sea cucumber called *Holothuria leucospilota* in an animal model of liver cancer caused by dimethyl nitrosamine (DMN) was studied.

**Methods:**

Wistar female rats were randomly divided into five groups (*n* = 12): control (intact), positive control (received 1% DMN [10 mg/kg/week, intraperitoneally] for 12 weeks), and three treatment groups (received 50, 100, and 200 mg/kg/day *H. leucospilota* extract orally for 12 weeks along with intraperitoneal administration of 1% DMN [10 mg/kg/week]). In all groups, ultrasound was performed on the liver every week to check its density. Blood sampling and liver isolation were performed on three occasions, at 4, 8, and 12 weeks, to check liver enzymes and the histopathological condition of the liver tissue (every week, four animals from each group were randomly selected).

**Results:**

Liver density changes were evident from the eighth week onward in the positive control group. Histopathological results indicated pathologic changes in the positive control group after 4 weeks. The increase in liver enzymes in the positive control group was significantly different from that in the treatment and control groups.

**Conclusions:**

We demonstrated the hepatoprotective effect of *H. leucospilota* on DMN‐induced liver damage in rats using biochemical and histological parameters and ultrasonography. More additional research (in silico or in vitro) is needed to find the exact mechanism and the main biological compound in *H*. *leucospilota*.

## Graphical abstract

Wistar female rats were randomly divided into five groups (*n* = 12): control (intact), positive control (received 1% DMN [10 mg/kg/week, intraperitoneal] for 12 weeks), and three treatment groups (received 50, 100, and 200 mg/kg/day *Holothuria leucospilota* extract orally for 12 weeks along with intraperitoneal administration of 1% DMN [10 mg/kg/week]). In all groups, a liver ultrasound was performed every week to check the density of the liver. Blood sampling and liver isolation were performed on three occasions, 4, 8, and 12 weeks, to check liver enzymes and the histopathological condition of the liver tissue (every week, four animals from each group were randomly selected).

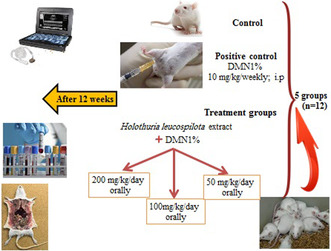

## 1 INTRODUCTION

One of the most common cancers with a poor prognosis worldwide is liver cancer.^1^ Among primary malignant tumors of the liver, hepatocellular carcinoma (HCC) is the most common. HCC is characterized by distinctive features like hidden development, poor diagnosis, and high mortality.^2^ The World Health Organization estimates that ~900 000 people worldwide are diagnosed with HCC, the sixth most common type of cancer in both sexes. HCC is the third most common cause of cancer‐related deaths worldwide: more than 830 000 people die from HCC each year. HCC mortality rates are higher in Asia and Africa than in any other continent.^3^ It was reported that the 5‐year survival rate of HCC is less than 20%, although it depends on the stage of diagnosis and treatment.^4^ Now, oncologists have concluded that cancer may occur via environmental factors such as dimethyl nitrosamine (DMN) that primarily targets hepatocytes.^5^ Hepatocytes contain a microsomal membrane‐bound enzyme, cytochrome P‐450IIE1, for DMN metabolic activation and degradation. Formaldehyde and methanol are produced upon DMN degradation in the liver, and subsequently alkylating intermediates may react in particular with susceptible macromolecules like nucleic acids and proteins to form methylated products.^6^

Given that anticancer drugs, besides the side effects, have relatively short half‐lives in the body,^7^ there is an effort to replace them with safer and prolonged‐effect compounds. Of the various kinds of research on introducing anticancer agents, natural products have gained attention from scientists. Sea cucumbers are a diverse group of marine invertebrates that live in warm shallow water, generally near corals, weeds, and rocks; they have elongated and worm‐like bodies, resembling cucumbers.^8^ These organisms are traditionally considered food, particularly among the Asian population. Dehydrated sea cucumbers are sold in markets, especially in China, Korea, Indonesia, and Japan.^9^, ^10^ It is a marine source with high nutritional and medicinal values in Asian folk medicine. The medicinal properties of these animals can be associated with a wide range of functional components with various biological activities.^8^ There are a wide range of biological effects such as antifungal, antimicrobial, antihemolytic, and antitumor properties in saponins and glycosides of sea cucumber. In addition, the anticancer properties of oligosaccharides and triterpenes of sea cucumber species have previously been reported in HeLa cell lines.^11^ Glycoprotein extracted from a species of sea cucumber can inhibit the proliferation of mouse S180 sarcoma cancer cells.^12^

We investigated the hepatoprotective effect of the methanolic extract of a species of sea cucumber called *Holothuria leucospilota* in an animal model of liver cancer induced by DMN.

## 2 MATERIALS AND METHODS

### 2.1 Chemicals

DMN was purchased from Sigma‐Aldrich (Saint Louis, MO, USA). Methyl alcohol, Entellan glue, and ethyl alcohol 99.6% were supplied by Merck (Gernsheim, Germany). Xylene and ethyl alcohol 50%–96% were purchased from Shimi‐lab (Tehran, Iran).

### 2.2 Sampling, identification of sea cucumber, and preparation of the extract

*H. leucospilota* was obtained from Hamoon Jetty, Qeshm Island, and the Persian Gulf, Iran. After morphological confirmation and removal of the internal organs, the samples were packed with ice, transferred to the laboratory, and finally stored at −80°C. For identification, small pieces of dorsal and ventral cuticles were cut and placed on a glass microscope slide with 2 mL of NaOCl until the soft tissues completely dissolved and ossicles in the form of white pellets appeared in the liquid. Ossicles were examined under a light microscope and confirmed using identical keys.^13^, ^14^, ^15^

The body surface of *H. leucospilota* was cleaned, cut into small pieces, and freeze dried. Then, 100 g of freeze‐dried samples were used for extraction with methanol in two separate steps of 48 and 24 at room temperature. After having removed solid residue by filtration and centrifugation (15 min, 30 000 × *g*, 4°C), the solvents were concentrated using a rotary evaporator at 30°C and dried using a freeze dryer (Christ, Alpha 2‐4 LD plus, Gröbenzell, Germany) to produce a solid form of methanolic extract (25 g).

### 2.3 Animals and diet

Female Wistar rats (weight: 220 ± 10 g, age: 6 weeks) were obtained from the Institute Pasteur of Iran and maintained in the Laboratory Animal Centre of Shiraz University (Fars, Iran). Animals were housed in an environment at a temperature of 20–22°C, a humidity of 50% ± 5%, and a 12‐h light–dark cycle, and the animals had free access to food and water. The ingredients in rat feed pellets included cereal grains, vegetable protein meals, legumes, vegetable oil, calcium carbonate, mono‐di calcium phosphate, salt, vitamin and mineral premix, and essential amino acids.

### 2.4 Dose determination and toxicity study

The acute oral toxicity of *H. leucospilota* extract was evaluated in rats using the limit dose test of the up and down procedure, test guideline 425 (OECD 425).^16^ It has been proved that *Holothuria* species are nontoxic in animal models.^17^ We decided to start animal dosing at the limited dose of 2000 mg/kg. The weights of animals were recorded before and after being subjected to *H. leucospilota* extract. The clinical signs of toxicity, behavioral changes, any abnormalities in the fur of skin, mucosal discharges, autonomic and central nervous system changes, and respiratory and heart rates of animals were controlled for 14 days, periodically.^16^ Finally, the animals were killed by CO_2_ anesthesia, and blood samples were obtained. Gross and histopathological examinations of internal organs were performed.

### 2.5 Experimental design

After acclimatization for 2 weeks, the rats were randomly divided into five groups (*n* = 12) as follows:

- Group 1: control (intact).
- Group 2: positive control (received 1% DMN [10 mg/kg/week, intraperitoneal (i.p.)] for 12 weeks).
- Groups 3–5: treatment groups (received 50, 100, and 200 mg/kg/day *H. leucospilota* extract orally for 12 weeks along with administration of 1% DMN [i.p., 10 mg/kg/week]).

The process of induced changes in rat liver by test compounds was evaluated weekly using Portable Ultrasound (GE Voluson 730 Pro) and linear transducer with a frequency of 13.5 MHz. At the end of the treatment, the animals were not fed for 24 h before they were killed by CO_2_ anesthesia.

### 2.6 Liver ultrasound examination

To investigate the changes in the liver after the administration of DMN in living rats, the ultrasound technique was applied weekly; we employed a portable ultrasound device (Volosun 730 G.E. PRO) and a 13.5‐MHz‐frequency linear transducer. After having anesthetized the animals and removed the fur of the animal's abdominal area, an ultrasound was performed from below the chest.

### 2.7 Blood sample analysis

The animals were not fed for 12 h on the last day before the induction of anesthesia or the collection of blood samples.

Blood samples were collected by cardiac puncture in terminal anesthesia conditions for analyzing the liver function tests, including aspartate transaminase (AST), alanine transaminase (ALT), alkaline phosphatase (ALP), and lactate dehydrogenase (LDH) using an Exigo analyzer (Boule Medical AB, Stockholm, Sweden) and commercially available diagnostic laboratory kits (Biorexfars).

Serum AST and ALT levels were measured according to the International Federation of Clinical Chemistry and Laboratory Medicine guidelines.^18^ AST was measured based on the photometric method. On addition of the reagent, serum aminotransferases convert the amino acid to the corresponding α‐ketoacid (glutamate). Therefore, the level of this compound indicates the activity of aspartate aminotransferase in the sample. Then oxaloacetate is indirectly converted to the corresponding hydroxyl acids (malate) by reduction by malate dehydrogenase.

ALT was measured based on the photometric method. On addition of the reagent, aminotransferases in the serum convert amino acid alanine to the corresponding α‐ketoacid (pyruvate). The level of this compound indicates the activity of alanine aminotransferase. In the process of the pyruvate reaction, it is indirectly converted to the corresponding hydroxyl acids (l‐lactate) by reduction by LDH. ALP was measured based on the photometric method; this enzyme hydrolyzes *p*‐nitrophenol phosphate to *p*‐nitrophenol in an alkaline environment, in di‐ethanolamine buffer, magnesium, and zinc ions.

LDH was also measured based on the photometric method; this enzyme leads to the reversible oxidation of l‐lactate to pyruvate; this reaction is mediated by the hydrogen acceptor, NAD+, and is the basis for measuring LDH activity.

### 2.8 Histopathological studies

To examine the histopathological conditions, tissue samples were rapidly removed from the left lateral lobe of the liver parenchyma, rinsed in cold saline, and immediately fixed by immersion in 10% neutral buffered formalin. Then, the fixed tissue specimens were dehydrated in a series of graded alcohol solutions, cleared in xylene, and embedded in paraffin wax. Liver tissue sections (5 μm thick) were mounted on slides stained with hematoxylin and eosin (H&E) and finally examined under a light microscope (Olympus, Tokyo, Japan).

### 2.9 Statistical analysis

IBM SPSS Statistics for Windows, version 21.0 (IBM Corp., Armonk, NY, USA), was used to analyze the obtained data. After the normal distribution of data was checked, they were subjected to one‐way analysis of variance (ANOVA) and then Tukey's post hoc test to determine the differences among groups. The level of significance was considered *p <* 0.05.

## 3 RESULTS

### 3.1 Identification of *H. leucospilota*

**FIGURE 1:**
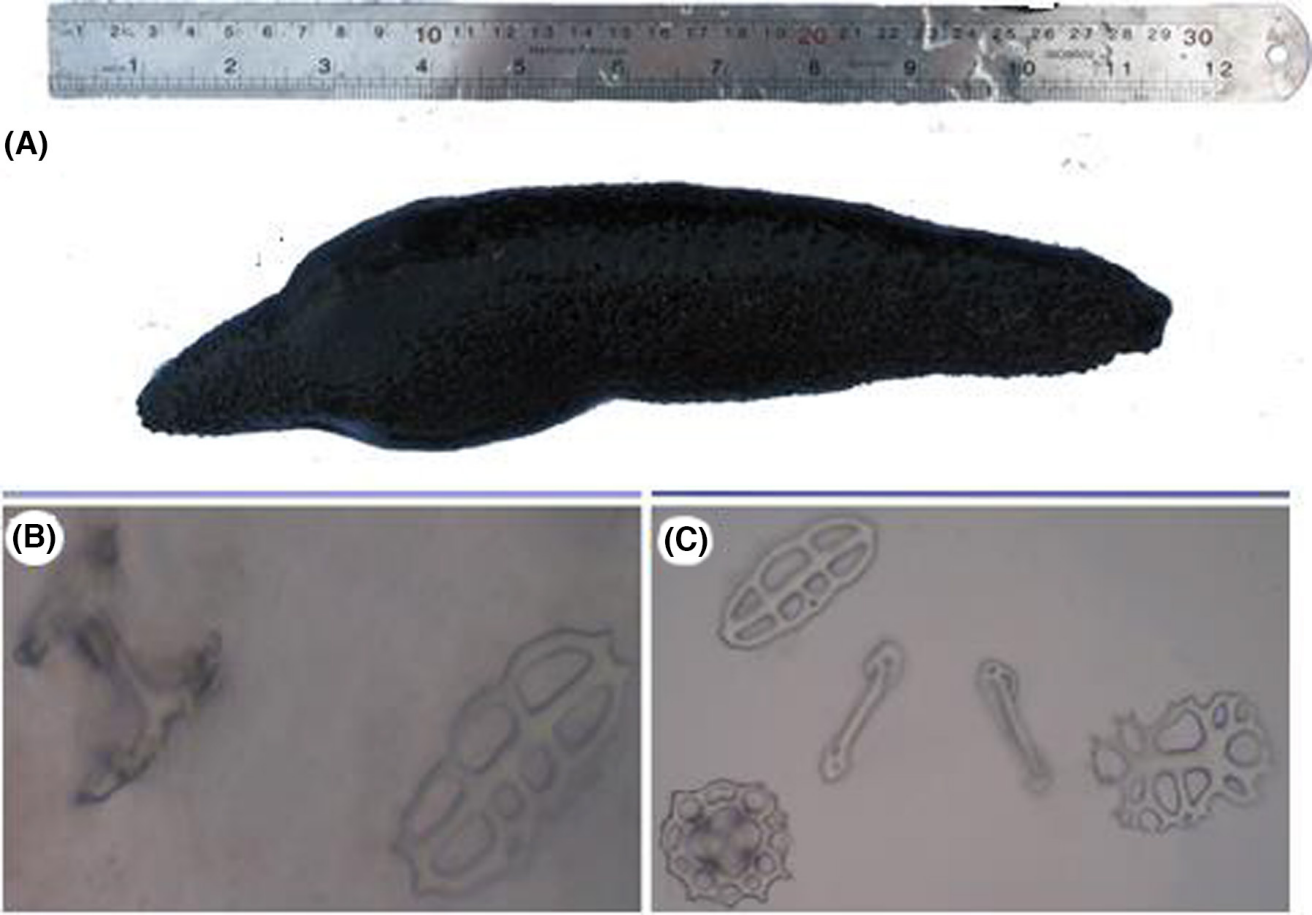
(A) *Holothuria leucospilota* dorsal view; (B) ossicle shapes in the dorsal cuticles and (C) in the ventral cuticles of *H. leucospilota*.

The body of *H. leucospilota* is narrow and cylindrical. The front part is stretched from the rear. The body is quite dark, and it has a smooth coating. Spicules of the dorsal body wall are button‐ and table shaped. Spicules of the ventral body wall are button‐, anchor‐, and table shaped (Figure 1).

### 3.2 Dose determination and toxicity study

Our results revealed that 2000 mg/kg of *H. leucospilota* extract was not toxic. None of the five rats died or showed any sign of toxicity at this dose. Therefore, median lethal dose (LD_50_) was considered above 2000 mg/kg of extract orally. The median effective dose was selected as one‐tenth, one‐twentieth, and one‐fortieth of the proposed LD_50_ (50, 100, and 200 mg/kg).

### 3.3 Liver ultrasound results

**FIGURE 2:**
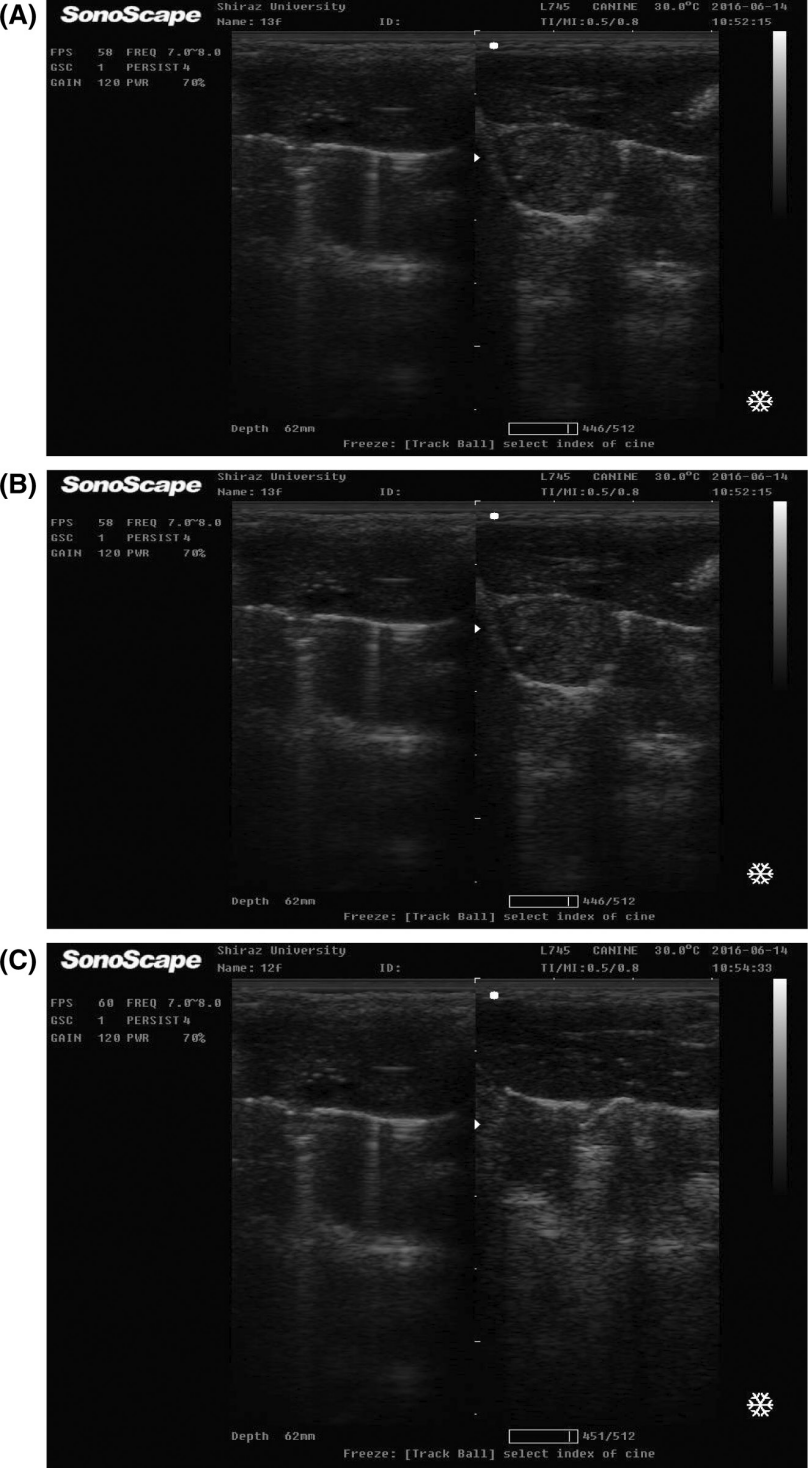
Comparison of liver graphs of tested rats after ultrasound in the last week of the experiment; positive control group; (A) Multiple hypoechogenic solid masses are visible in the liver; nodules with a typical lateral shadow are detected in the positive group; (B) A hypoechoic lesion is observed in the liver and demonstrated vascularity on Doppler study; control intact; (C) Normal echotexture and hypoechogenic of liver.

Until the fourth week, the density and echogenicity of the liver in all groups were similar to those in the control group. From the 7th week in the positive control group and the 10th week in the other groups, liver tissue changes were observed in the form of a hypoechoic liver area, which indicates a decrease in the integrity of the liver tissue. Dense radiopaque areas (indicating tissue change) were observed in none of the treatment groups. From the 8th to the 12th weeks, hyperechoic areas were observed in the positive control group (Figure 2; Table 1).

**TABLE 1:** Changes in liver consistency during 12 weeks among all groups using ultrasound.

Week group	1	2	3	4	5	6	7	8	9	10	11	12
Control	$\frac{0}{12}$	$\frac{0}{12}$	$\frac{0}{12}$	$\frac{0}{12}$	$\frac{0}{8}$	$\frac{0}{8}$	$\frac{0}{8}$	$\frac{0}{8}$	$\frac{0}{4}$	$\frac{0}{4}$	$\frac{0}{4}$	$\frac{0}{4}$
Positive control	$\frac{0}{12}$	$\frac{0}{12}$	$\frac{0}{12}$	$\frac{0}{12}$	$\frac{0}{8}$	$\frac{0}{8}$	$\frac{0}{8}$	$\frac{1}{8}$	$\frac{1}{4}$	$\frac{2}{4}$	$\frac{4}{4}$	$\frac{3}{3}$
Treatment 50 mg/kg	$\frac{0}{12}$	$\frac{0}{12}$	$\frac{0}{12}$	$\frac{0}{12}$	$\frac{0}{8}$	$\frac{0}{8}$	$\frac{0}{8}$	$\frac{0}{8}$	$\frac{0}{4}$	$\frac{0}{4}$	$\frac{0}{4}$	$\frac{0}{4}$
Treatment 100 mg/kg	$\frac{0}{12}$	$\frac{0}{12}$	$\frac{0}{12}$	$\frac{0}{12}$	$\frac{0}{8}$	$\frac{0}{8}$	$\frac{0}{8}$	$\frac{0}{8}$	$\frac{0}{4}$	$\frac{0}{4}$	$\frac{0}{4}$	$\frac{0}{4}$
Treatment 200 mg/kg	$\frac{0}{12}$	$\frac{0}{12}$	$\frac{0}{12}$	$\frac{0}{12}$	$\frac{0}{8}$	$\frac{0}{8}$	$\frac{0}{8}$	$\frac{0}{8}$	$\frac{0}{4}$	$\frac{0}{4}$	$\frac{0}{4}$	$\frac{0}{4}$

### 3.4 Histopathological results of liver

**FIGURE 3:**
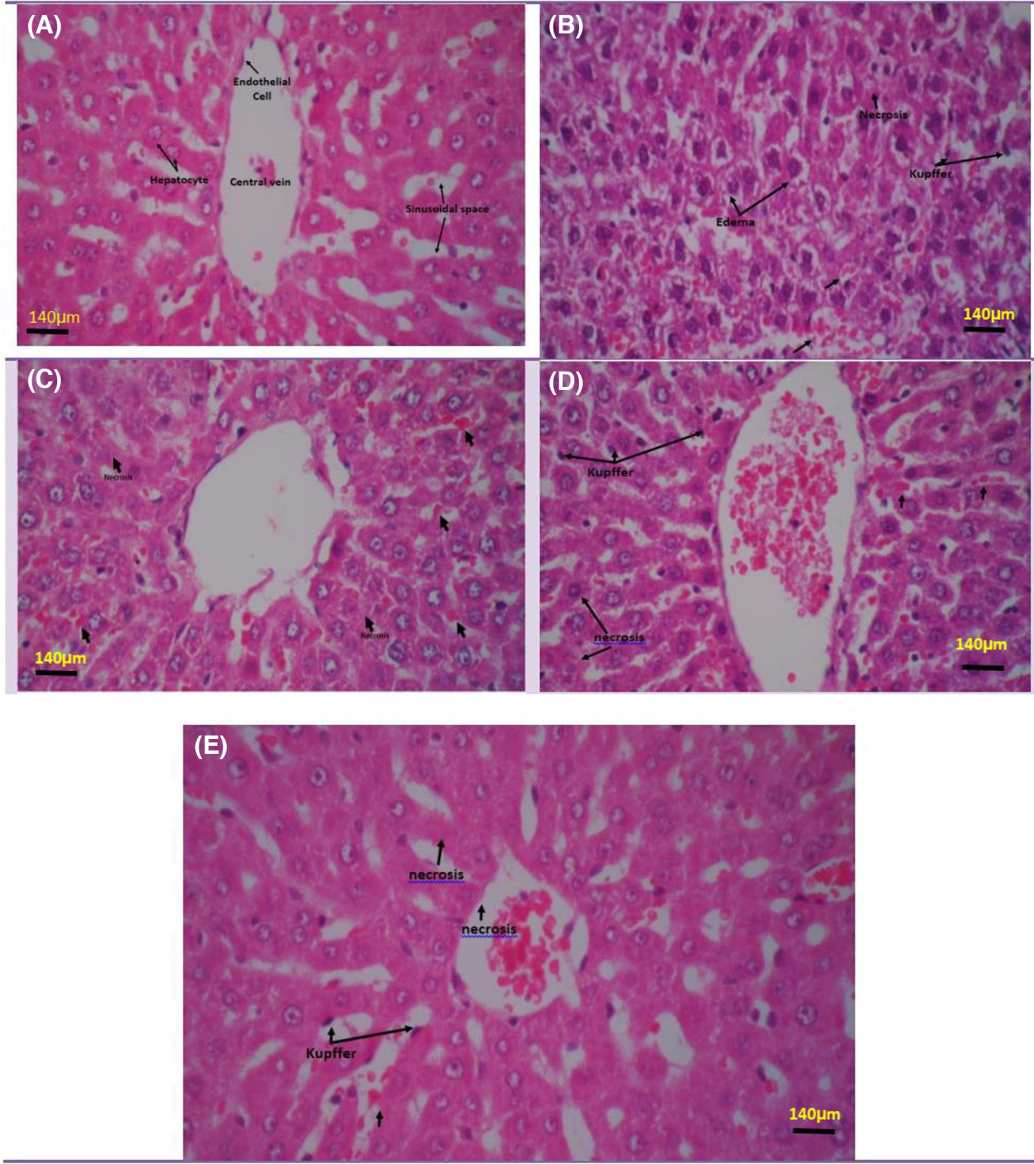
Microimages of hematoxylin–eosin‐stained sections of rat liver with and without the effect of sea cucumber extracts after induction of hepatotoxicity. (A) Control group; (B) positive control group, induced liver toxicity with dimethyl nitrosamine, hepatocytes with small and compact nuclei, and hyperemia and accumulation of inflammatory cells in the sinusoidal spaces; (C–E) histopathological changes in the treatment groups (25, 50, and 100 mg/kg), compared to the positive control group, significantly improved in a dose‐dependent manner (magnification: 400×).

The H&E staining technique revealed normal hepatocytes in the control group characterized by regular hexagonal lobules with terminal hepatic venules in the central structure and portal triads in the periphery. None of the treatment groups showed disorganization in the hepatic parenchyma and hepatocellular changes. The positive control group revealed a loss of structure and proliferative and neoplastic changes. Also, mild‐to‐moderate nuclear and cellular pleomorphism, increased nuclear‐to‐cytoplasmic ratio, hyperchromatism as well as conspicuous nucleoli, and mild vascular invasion of tumor cells were detected in this group. The mentioned characteristics represent a well‐differentiated HCC with a marked increase in the number of hepatocytes, an increase in the thickness of the hepatic plates, a narrowing of the sinusoidal spaces, and a fading of the hepatic sinusoids. In the treatment groups, histopathological lesions gradually improved with increasing extract doses (Figure 3).

### 3.5 Liver function test results

There was a significant increase in serum levels of ALT, AST, ALP, and LDH in the positive control group compared to the control and treatment groups (*p* < 0.05).

The results obtained in the fourth week of the study showed that the level of AST, ALT, ALP, and LDH in the positive control group, without treatment, was higher than that in the intact control group and treatment groups, and there was a statistically significant difference between these groups. Also, regarding the AST test, there was a statistically significant difference between the minimum‐dose (25 mg/kg) and the maximum‐dose (200 mg/kg) treatment groups. These results show that liver damage started in untreated animals from the first week of the study. On the contrary, the results of impeding progress in liver damage in the treatment groups indicate that the effect of sea cucumber extract is dose dependent (Table 2). The results in the eighth week were similar to those in the fourth, with a statistically significant difference in the ALT test in the treatment groups so that the maximum‐dose treatment group (200 mg/kg) had a significant difference compared with the other treatment groups. These results show that with the progression of liver damage, higher doses of sea cucumber performed better in controlling the damage. In the 12th week of the study, the AST, ALT, ALP, and LDH tests in the treatment and control groups showed statistically significant differences with the positive control group. On the contrary, the treatment groups exhibited dose‐dependent characteristics in ALT and ALP tests, so the maximum dose (200 mg/kg) was better than the minimum dose (25 mg/kg) in controlling liver damage (Table 2).

**TABLE 2:** The mean ± SD of liver function enzymes in different groups of rats in the 4th, 8th, and 12th weeks.

Time (week)	Groups	LDH (U/mL) Mean ± SD	ALP (U/mL) Mean ± SD	AST (U/mL) Mean ± SD	ALT (U/mL)Mean ± SD
4	Control	1024 ± 11^a^	351 ± 29^a^	117 ± 7^a^, ^b^	38 ± 8^a^
	Positive control	2535 ± 229	635 ± 15	175 ± 3	83 ± 3
	Treatment 25 mg/kg	1105 ± 7^a^	393 ± 7^a^	125 ± 2.5^a^	48 ± 3^a^
	Treatment 100 mg/kg	1074 ± 8^a^	377 ± 4^a^	122 ± 3^a^	44 ± 3^a^
	Treatment 200 mg/kg	1074 ± 12^a^	372 ± 5^a^	115 ± 3^a^, ^b^	40 ± 2^a^
8	Control	1028 ± 14^a^	341 ± 22^a^	112 ± 7^a^	36 ± 4^a^, ^b^, ^c^
	Positive control	3277 ± 279	745 ± 51	196 ± 12	93 ± 4
	Treatment 25 mg/kg	1260 ± 37^a^	420 ± 9^a^	130 ± 4^a^	54 ± 4^a^
	Treatment 100 mg/kg	1144 ± 21^a^	387 ± 7^a^	123 ± 4^a^	51 ± 4^a^
	Treatment 200 mg/kg	1112 ± 23^a^	387 ± 8^a^	120 ± 2^a^	41 ± 2^a^, ^b^, ^c^
12	Control	1031 ± 19^a^	353 ± 2^a^	110 ± 5^a^	37 ± 3^a^, ^b^
	Positive control	4224 ± 336	880 ± 62	293 ± 131	112 ± 5
	Treatment 25 mg/kg	1511 ± 42^a^	470 ± 8^a^	137 ± 2^a^	59 ± 1^a^
	Treatment 100 mg/kg	1361 ± 36^a^	432 ± 5^a^	130 ± 1^a^	55 ± 2.5^a^
	Treatment 200 mg/kg	1275 ± 34^a^	420 ± 5^a^, ^b^	123 ± 1^a^	44 ± 3^a^, ^b^

Abbreviations: ALP, alkaline phosphatase; ALT, alanine transaminase; AST, aspartate transaminase; LDH, lactate dehydrogenase; SD, standard deviation.

^a^ Significant difference with the positive control group (*p* < 0.001).

^b^ Significant difference with the treatment group (25 mg/kg) (*p* < 0.001).

^c^ Significant difference with the treatment group (100 mg/kg) (*p* < 0.001).

### 3.6 Animal weight monitoring results

**FIGURE 4:**
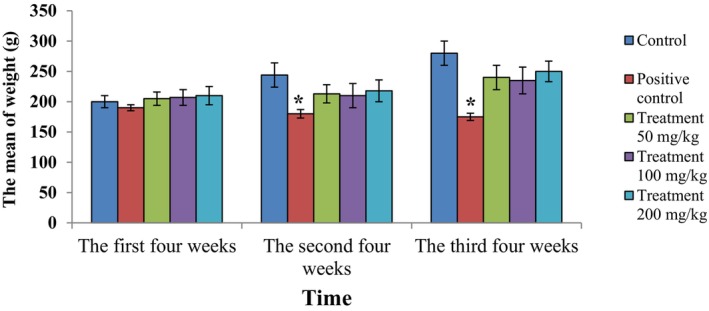
The mean weight changes in animals in various groups every 4 weeks for 12 weeks. *Significant weight loss after 8 weeks in the positive control group compared to the control group (*p* < 0.05).

The mean weight changes in animals in various groups were monitored every 4 weeks for 12 weeks. The results showed a significant weight loss after 8 weeks in the positive control group compared to the other groups (*p* < 0.05), whereas no statistically significant difference in weight was observed in the remaining groups (Figure 4).

## 4 DISCUSSION

In this observational study, we investigated the hepatoprotective effect of *H. leucospilota* extract on HCC induced in rat animal models by DMN. Our study showed that *H. leucospilota* is nontoxic up to 2000 mg/kg, and 25, 100, and 200 mg/kg used in this study could improve the toxic effects of DMN in the liver of rats. The results of this study were confirmed by an ultrasound of the ventricular area and changes in liver density, liver function enzymes, and histopathology.

DMN belongs to the group of nitrosamine compounds (R1N (−R2)–N=O), in which the nitroso group bonds to an amine. Nitrosamine compounds are mainly found in cured meats, smoked fish, and cigarette smoke.^19^ Most nitrosamines, including DMN, are carcinogens and cause liver fibrosis in the short term and liver cancer in the long term. DMN is a yellow and inactive liquid that turns into active metabolites after entering the hepatocytes; the binding of DMN to the DNA of hepatocytes causes DNA alkylation at bases and the phosphate group. Alkylation of oxygen atoms in DNA bases is essential for the carcinogenic effect of DMN.^20^

HCC is among the most common liver cancers in the world. Alcohol consumption, viral infections, aflatoxin, nonalcoholic fatty liver, Wilson's disease, type 2 diabetes, and hemophilia are among the most risk factors of HCC.^21^

*H. leucospilota* is commonly found in shallow water (<10 m) on outer and inner reefs, back reefs, and shallow coastal lagoons. Shallow‐water species are denser, composed of smaller sections, and reproduce mostly by transversal fission. In comparison, in deeper or outer reef species the sections are more scattered and more prominent; their reproducing systems involve two sexes.^22^ *H. leucospilota* is the prevalent sea cucumber species in the Persian Gulf.^23^ Our study revealed that *H. leucospilota* extract was not toxic at 2000 mg/kg. None of the five rats died or showed any sign of toxicity at this dose. In compliance with our toxicological study on these sea cucumbers, other *Holothuria* species, *Holothuria atra* and *Holothuria arenicola*, are nontoxic at 2000 mg/kg in animal models.^17^, ^24^

Previous studies have also discussed the anticancer effects of sea cucumbers of the genus *Holothuria*. Zhang et al. evaluated the effects of *Holothuria fuscocinerea* species, which led to the isolation of three new types of triterpene glycosides; pericoside C; two types of glycosides; fuscocineroside A, B, and C; and cyhalothrin A, which have cytotoxic effects. The effect of this species on cancer cell lines was attributed to the effective compounds present in this species of sea cucumber.^25^ On the contrary, the remarkable antitumor effect of triterpene glycosides, quadrangularisosides A, C, E, and F, has been reported from the sea cucumber *Colochirus quadrangularis*.^26^, ^27^ Chemotherapy studies have shown a synergistic effect of the triterpene glycoside, frondozide A, obtained from the Atlantic sea cucumber, *Cucumaria frondosa*.^28^ *Holothuria (Halodeima) atra* also exhibited effective antitumor and antifungal activities.^29^ In previous research, the cytotoxic effect of ethyl acetate extract of *H. leucospilota* and *Holothuria parva* was determined.^30^ Sea cucumber contains philinopside E, which can inhibit the proliferation of human umbilical vein endothelial cells and human microvascular endothelial cells. On the contrary, <2 μmol/L of this compound induced apoptosis in the mentioned cell lines.^31^ A sulfated saponin is a group of compounds extracted from sea cucumbers that can inhibit angiogenesis around the tumor. Also, the cytotoxic effect of *Mensamaria intercedens* on mouse S180 sarcoma and mouse Lewis lung cancer was attributed to three triterpene glycosides, intercedenside A, B, and C, isolated from this cucumber species.^12^

Various characteristics of HCC in ultrasound such as small and typically hypoechoic lesions or larger lesions with mixed echogenicity or hyperechoic lesions may exist. An abnormal ultrasound pattern was observed only in the positive control group, which identified changes in liver consistency during weeks 8–12. Although ultrasound‐based techniques have been reported as sensitive techniques for detecting abnormalities in the liver, this method is not effective in detecting lesions in mild diseases, so the accuracy of diagnosis is based on the histopathologic method.^32^ In the present study, the failure of sonography to distinguish between normal hepatic parenchyma and early changes in rat liver might help explain the previously reported findings on the inoperative application of ultrasound to mild hepatic changes. In addition, we confirmed liver damage from liver function tests (ALT, AST, ALP, and LDH) among groups. The mean of serum ALT, AST, ALP, and LDH significantly increased in the positive control group compared to the control and treatment groups. During tumor induction, the fluctuation in these enzymes may serve as one of the good screening biomarkers of HCC response to therapy.^33^ After hepatic cell damage, some tissue enzymes like ALT and AST are discharged from the cytoplasm into the blood.^34^ The activity of LDH increases in cell necrosis conditions, and ALP increases in cases of tumor cell differentiation.^35^ The more favorable results obtained from the maximum dose of sea cucumber in our study compared to other doses indicated that sea cucumber acts in a dose‐dependent manner in controlling liver damage caused by DMN.

In the present study, treatment with sea cucumber extracts indicated a statistically significant decrease in enzyme activities compared to the positive control group. The use of sea cucumbers in traditional Chinese medicine has yielded significant results in controlling liver cancers. In agreement with our study, acid mucopolysaccharide extracted from sea cucumber *Stichopus japonicas* exhibited an antitumor and preventive effect on HCC induced by DMN in rats.^33^

Although ultrasound diagnosis along with histopathological diagnosis can be helpful, sometimes immunohistochemical staining (IHC) is very useful for definitive diagnosis, especially when the sampling volume is small. IHC markers for HCC diagnosis include hepatocellular differentiation—such as hepatocyte paraffin 1 and arginase‐1—and those of malignant hepatocytes—such as glypican‐3, heat shock protein 70, and glutamine synthetase.^36^ One of the limitations of our study was the failure to investigate immunohistochemical markers related to HCC, which should be considered in future research. On the contrary, new strategies for auxiliary diagnosis and prognosis of HCC have been introduced, which include the examination of potential serum tumor markers, such as α‐fetoprotein, Golgi protein 73, phosphatidylinositol proteoglycan, osteopontin, abnormal prothrombin, and heat shock protein.^37^ We did not investigate the mentioned serum markers due to the lack of access to the relevant commercial kits, which can also be considered in future research.

## 5 CONCLUSIONS

This study investigated the effect of sea cucumber extract of *H. leucospilota* on liver damage caused by DMN based on biochemical, histopathological, and ultrasound results. We showed that a 12‐week administration of *H. leucospilota* extract can control liver damage caused by DMN. The results of this study were confirmed based on the level of liver enzymes, histopathology results of liver samples, and ultrasound examination.

Considering that *H. leucospilota* has therapeutic properties, it is suggested that it should be reexamined after identifying its effective compounds in improving liver damage. If a favorable response is obtained, studies can also be performed at the clinical level.

## AUTHOR CONTRIBUTIONS

All authors contributed to the conception and design of this study. Fatemeh Dejan: literature search, laboratory work, data acquisition, data analysis, manuscript preparation, manuscript editing, manuscript review; Javad Rajabi Aslani: literature search, laboratory work, data acquisition, data analysis, manuscript preparation, manuscript editing, manuscript review; Hamid Reza Gheisar: concepts, design, definition of intellectual content, clinical studies, guarantor; Narges Eskandari Roozbahani: clinical studies, manuscript preparation, manuscript editing, manuscript review; Amineh Daneshi: data acquisition, statistical analysis, manuscript preparation, manuscript review; Reza Behmanesh: radiology and ultrasonography, manuscript review; Elaham Rahmanian and Nasrollah Ahmadi: laboratory work, pathology and preparation of tissue slides, manuscript review.

## FUNDING INFORMATION

This work was supported by Shiraz University, Shiraz, Iran. Hamid Reza Gheisari has received research support from Shiraz University.

## CONFLICT OF INTEREST STATEMENT

The authors have no conflicts of interest to declare.

## ETHICS APPROVAL AND CONSENT TO PARTICIPATE

Procedures adopted in this study followed Shiraz University's ethical guidelines for use of animals in experimental studies that were compatible with Helsinki ethics guidelines. None of the authors mentioned in this article have a purpose or duty other than research and/or education. This research has neither financial nor spiritual support from the government.
